# Machine learning is an effective method to predict the 3-month prognosis of patients with acute ischemic stroke

**DOI:** 10.3389/fneur.2024.1407152

**Published:** 2024-06-12

**Authors:** Qing Huang, Guang-Li Shou, Bo Shi, Meng-Lei Li, Sai Zhang, Mei Han, Fu-Yong Hu

**Affiliations:** ^1^School of Public Health, Bengbu Medical University, Bengbu, Anhui, China; ^2^Department of Neurology, The Second Affiliated Hospital, Bengbu Medical University, Anhui, China; ^3^School of Medical Imaging, Bengbu Medical University, Anhui, China; ^4^Department of Emergency Medicine, The Second Affiliated Hospital, Bengbu Medical University, Anhui, China

**Keywords:** ischemic stroke, machine learning, prognosis, prediction model, random forest

## Abstract

**Background and objectives:**

Upwards of 50% of acute ischemic stroke (AIS) survivors endure varying degrees of disability, with a recurrence rate of 17.7%. Thus, the prediction of outcomes in AIS may be useful for treatment decisions. This study aimed to determine the applicability of a machine learning approach for forecasting early outcomes in AIS patients.

**Methods:**

A total of 659 patients with new-onset AIS admitted to the Department of Neurology of both the First and Second Affiliated Hospitals of Bengbu Medical University from January 2020 to October 2022 included in the study. The patient’ demographic information, medical history, Trial of Org 10,172 in Acute Stroke Treatment (TOAST), National Institute of Health Stroke Scale (NIHSS) and laboratory indicators at 24 h of admission data were collected. The Modified Rankine Scale (mRS) was used to assess the 3-mouth outcome of participants’ prognosis. We constructed nine machine learning models based on 18 parameters and compared their accuracies for outcome variables.

**Results:**

Feature selection through the Least Absolute Shrinkage and Selection Operator cross-validation (Lasso CV) method identified the most critical predictors for early prognosis in AIS patients as white blood cell (WBC), homocysteine (HCY), D-Dimer, baseline NIHSS, fibrinogen degradation product (FDP), and glucose (GLU). Among the nine machine learning models evaluated, the Random Forest model exhibited superior performance in the test set, achieving an Area Under the Curve (AUC) of 0.852, an accuracy rate of 0.818, a sensitivity of 0.654, a specificity of 0.945, and a recall rate of 0.900.

**Conclusion:**

These findings indicate that RF models utilizing general clinical and laboratory data from the initial 24 h of admission can effectively predict the early prognosis of AIS patients.

## Introduction

The 2019 Global Burden of Disease Study findings reveal that stroke continues to be the second leading cause of death and ranks third in causing disability worldwide (1). Since 2015, stroke has emerged as the preeminent cause of death and disability in China, significantly impacting the nation’s public health and exerting substantial pressure on its healthcare infrastructure (2). Acute ischemic stroke (AIS), constituting over 80% of stroke cases (3), remains the predominant type. Statistics indicate that upwards of 50% of stroke survivors endure varying degrees of disability, with a recurrence rate of 17.7% (4), thereby placing a considerable strain on both families and society at large. Consequently, the early detection and prognosis of individuals at elevated risk of AIS, coupled with prompt intervention and treatment, hold critical importance in enhancing the quality of life for AIS patients.

Presently, studies on clinical prediction of ischemic stroke outcomes predominantly employ established scales or conventional models, such as the Essen stroke risk score, RRE-90 score, Logistic regression, and Cox regression (5–7). These tools, however, are limited by their inclusion of only a select few traditional risk factors and an inability to capture complex nonlinear interactions among various prognostic elements. In contrast, Machine Learning (ML)—a field exploring the simulation of human learning processes by computer algorithms (8)—holds distinct advantages. ML eschews the prerequisite assumptions about variable relationships and model structures inherent to traditional statistics and can adeptly manage datasets with incomplete entries. Additionally, ML models offer explanatory insights that surpass classical statistical methods (9). With the advent of big data analytics in healthcare research, machine learning is integrating increasingly vast data arrays to more accurately reflect human physiological complexity and the unpredictable aspects of disease traits. This progression heralds an innovative trajectory for disease diagnosis, prognosis, and risk evaluation, offering substantial practical application potential (10–12).

In this study, we develop and validate a predictive model for the early prognosis of patients experiencing their first AIS using relevant medical data. We employ various ML algorithms to assess the efficacy of the model and compare the predictive utility of each to determine the optimal mathematical model for forecasting early outcomes in AIS patients. The goal is to facilitate the identification of high-risk individuals with potentially poor prognoses at an early stage, thereby providing a scientific basis for selecting appropriate clinical treatment strategies.

## Objects and methods

### Subjects

Our study encompassed 659 patients who experienced their first AIS and were admitted to the Department of Neurology at both the First and Second Affiliated Hospitals of Bengbu Medical University from January 2020 to October 2022. The inclusion criteria mandated that (1) the disease presented within 2 weeks of onset, and (2) all participants must conform to the ischemic stroke diagnostic guidelines as revised at the Fourth National Academic Conference on Cerebrovascular Disease, with this incident being their initial occurrence confirmed via CT scan. The exclusion criteria excluded patients with (1) incomplete medical records, (2) CT or MRI evidence of intracranial hemorrhage, expansive infarctions across multiple lobes, tumors, or vascular malformations, (3) severe concurrent conditions involving the cardiac, hepatic, or renal systems, along with malignancies, (4) a history of significant cranial trauma or neurosurgery, and (5) coexisting autoimmune diseases, hematologic disorders, or severe infectious diseases. Ethical endorsement for this research adhered to the Declaration of Helsinki and was secured from the Medical Ethics Committee of Bengbu Medical University. Informed consent was obtained from the patients for this study.

### Data collection

Data on patients were gathered via an electronic case system, comprising (1) demographic information such as age and gender; (2) medical history, including high blood pressure, type II diabetes, coronary heart disease, atrial fibrillation; (3) TOAST etiologic classification of stroke; and (4) scores on the National Institute of Health Stroke Scale (NIHSS) (13) upon admission. Additionally, laboratory test results within 24 h of admission featured routine blood parameters like white blood cell (WBC), platelet (PLT), neutrophil (Neu), lymphocyte (Lym), monocyte (Mon), red blood cell (RBC) counts, and hemoglobin (HGB) concentration. Coagulation markers assessed were prothrombin time (PT), PT ratio (PTR), international normalized ratio (INR), activated partial thromboplastin time (APTT), clotting time (TT), fibrinogen (FIB), D-dimer, fibrinogen degradation product (FDP), and others. The analysis also included total cholesterol (CHOL), triacylglycerol (TG), low-density lipoprotein (LDL), high-density lipoprotein (HDL), uric acid (UA), C-reactive protein (CRP), homocysteine (HCY), glucose (GLU), and essential thyroid indices such as thyroid-stimulating hormone (TSH), free tri-iodothyronine (FT3), free thyroxine (FT4), total tri-iodothyronine (T3), and total thyroxine (T4). Calculations for the Neutrophil-to-lymphocyte ratio (NLR), Platelet-to-lymphocyte ratio (PLR), and Monocyte-to-lymphocyte ratio (MLR) were also included, defined, respectively, as Neu/Lym, PLT/Lym, and Mon/Lym ratios.

### Prognostic assessment

Prognostic information on participants was collected via telephone or during outpatient follow-up 3-months post-treatment, employing the Modified Rankin Scale (mRS) to assess outcomes. The mRS scores range from 0 to 6, with 0 signifying no symptoms and 6 denoting death. Scores are detailed as follows: (1) reflects mild symptoms without significant disability, allowing independence in daily tasks; (2) represents slight disability, hindering the performance of previous tasks yet permitting self-managed daily activities; (3–5) indicate escalating levels of disability, requiring assistance to varying degrees, with 5 specifically denoting severe disability, paralysis, incontinence, and the need for constant care. A good prognosis is defined by an mRS score of ≤2, while scores >2 signal a poor prognosis (14).

### Machine learning modeling

Random forests were initially employed to impute missing values, after which the data were split into a training set and a test set in a 7:3 ratio. The training set was utilized for parameter calculation and model construction, whereas the test set was used to assess prediction accuracy. Feature selection within the training set was conducted using the Least Absolute Shrinkage and Selection Operator cross-validation (Lasso CV) method, enabling the inclusion of feature variables with non-zero coefficients into various algorithms—eXtreme Gradient Boosting (XGB), Logistic Regression (LR), LGBM Classifier, Random Forest Classifier (RF), Ada Boost Classifier (AdaBoost), Decision Tree Classifier (DT), Gradient Boosting Classifier (GBDT), Multi-layer Perceptron Classifier (MLP), and Support Vector Machine (SVM)—to develop prediction models for the early prognosis of AIS patients. Model parameters were refined through 10-fold cross-validation, enhancing model optimization for performance evaluation on the training set. The most effective model was identified, and its classification efficacy on the test set was assessed using metrics including Area Under the Curve (AUC), accuracy, sensitivity, specificity, and recall.

Data filling was conducted utilizing the “missForest ()” function within the “missForest” package of R software version 4.2.3. Feature filtering was performed using the Python machine learning library “scikit-learn” version 1.1.3. For the modeling, we used the “XGBoost” (15) (version 2.0.1), “lightgbm” (16) (version 3.2.1) and “scikit-learn” (17) (version 1.1.3) Python Packages. All development and validation of machine learning models were carried out using Python version 3.11.4.

### Statistical analysis

For measures that followed a normal distribution, information was presented as mean ± standard deviation (x̅ ± s), while for those that did not follow a normal distribution, information was presented as the median (interquartile range, *IQR*). Count data were expressed as frequency and percentage (%). The chi-square (*χ^2^*) test was utilized to compare count data between groups, whereas the *t*-test or Mann–Whitney *U* test was applied for measured data comparisons. All statistical analyses were conducted using *SPSS* version 19.0, with a significance level set at *α* = 0.05.

## Results

### General conditions of the study population

This study encompassed 659 patients experiencing their first-ever AIS (AIS), comprising 370 males and 289 females, with an average age of 68.34 ± 12.36 years. Three-months post-treatment, 507 patients displayed a favorable prognosis (modified Rankin Scale, mRS ≤ 2), while 152 patients fell into the poor prognosis category (mRS > 2), marking an early poor prognosis incidence of 23.07%. Table 1 presents the detailed clinical data of the subjects categorized into these two groups.

**Table 1:** Clinical feature comparison between the two outcome groups.

Variants	mRS ≤ 2 (*n* = 507)	mRS > 2 (*n* = 152)	*p*-value
Demographic features			
Age (year), median (*IQR*)	69.00 (58.00–77.00)	71.00 (61.00–80.75)	0.026
Sex			0.030
Female, *n* (%)	234 (46.15)	55 (36.18)	
Male, *n* (%)	273 (53.85)	97 (63.82)	
History of previous illnesses			
High blood pressure, *n* (%)			0.259
Yes	229 (64.89)	73 (59.87)	
No	278 (35.11)	79 (40.13)	
T2MD			0.535
Yes	229 (45.17)	73 (48.03)	
No	278 (54.83)	79 (51.97)	
Coronary heart disease, *n* (%)			
Yes	148 (29.19)	45 (29.61)	
No	359 (70.81)	107 (70.39)	
Atrial fibrillation, *n* (%)			0.556
Yes	128 (25.25)	42 (27.63)	
No	379 (74.75)	110 (72.37)	
Toast, *n* (%)			0.011
Atherosclerosis	128 (25.25)	51 (33.55)	
Cardiac thrombosis	25 (4.93)	10 (6.58)	
Small-artery occlusion type	116 (22.88)	35 (23.03)	
Other etiologies	38 (7.50)	1 (0.66)	
Unknown cause	200 (39.45)	55 (36.18)	
NIHSS score, median (*IQR*)	4.00 (3.00–7.00)	7.00 (3.25–12.00)	<0.001
Laboratory features at 24 h after admission			
Routine blood tests			
WBC (×10^9^/L), median (*IQR*)	6.41 (5.30–7.44)	7.07 (6.11–9.01)	<0.001
PLT (×10^9^/L), median (*IQR*)	204.00 (170.00–237.00)	200.50 (167.50–249.75)	0.577
Neu (×10^9^/L), median (*IQR*)	4.17 (3.34–5.40)	5.48 (3.74–7.46)	<0.001
Lym (×10^9^/L), median (*IQR*)	1.66 (1.31–2.09)	1.29 (0.91–1.88)	<0.001
Mon (×10^9^/L), median (*IQR*)	0.42 (0.34–0.53)	0.43 (0.32–0.56)	0.851
RBC (×10^12^/L), median (IQR)	4.51 (4.18–4.88)	4.50 (4.01–4.82)	0.038
HGB (g/L), median (*IQR*)	137.00 (125.00–150.00)	135.50 (121.00–146.00)	0.031
CRP (mg/L), median (*IQR*)	1.89 (0.80–3.40)	2.70 (0.93–6.82)	0.002
Coagulation indicators			
PT (s), median (*IQR*)	11.10 (10.60–11.70)	11.00 (10.60–11.60)	0.179
PTR (%), median (*IQR*)	0.96 (0.91–1.00)	0.94 (0.91–0.99)	0.159
INR, median (*IQR*)	0.96 (0.90–1.00)	0.94 (0.91–0.99)	0.434
APTT(s), median (*IQR*)	27.20 (25.80–28.60)	26.65 (25.13–28.10)	0.018
TT(s), median (*IQR*)	17.60 (17.00–18.10)	17.35 (16.70–17.91)	0.023
FBG (g/L), median (*IQR*)	2.84 (2.35–3.37)	3.15 (2.64–3.91)	<0.001
D-dimer (mg/L), median (*IQR*)	0.35 (0.23–0.65)	0.70 (0.33–1.60)	<0.001
FDP (ug/mL), median (*IQR*)	1.34 (0.80–2.30)	2.20 (1.15–4.66)	<0.001
Biochemical indicators			
CHOL (mmol/L), median (*IQR*)	4.50 (3.73–5.20)	4.52 (3.78–5.45)	0.438
TG (mmol/L), median (*IQR*)	1.43 (1.12–1.83)	1.39 (1.03–1.81)	0.303
LDL (mmol/L), median (*IQR*)	2.44 (2.00–2.99)	2.45 (1.97–3.43)	0.330
HDL (mmol/L), median (*IQR*)	1.23 (1.04–1.40)	1.18 (0.94–1.40)	0.122
UA (umol/L), median (*IQR*)	280.54 (248.00–328.71)	285.50 (222.50–349.00)	0.957
HCY (umol/L), median (*IQR*)	14.00 (11.46–17.00)	16.00 (13.00–22.15)	<0.001
GLU (mmol/L), median (*IQR*)	6.01 (5.10–7.60)	6.50 (5.20–6.50)	0.006
Thyroxine test indicators			
TSH (uIU/mL), median (*IQR*)	2.74 (1.90–3.55)	2.94 (1.89–3.48)	0.726
FT3 (pmol/L), median (*IQR*)	2.79 (2.64–3.26)	2.79 (2.59–3.21)	0.257
FT4 (pmol/L), median (*IQR*)	8.72 (8.20–9.89)	8.80 (8.26–11.12)	0.346
T3 (ng/mL), median (*IQR*)	1.04 (0.90–1.11)	1.045 (0.91–1.12)	0.829
T4 (ng/mL), median (*IQR*)	67.30 (58.50–72.63)	67.99 (55.33–75.43)	0.517
NLR, median (*IQR*)	2.46 (1.76–3.52)	4.25 (2.39–6.45)	<0.001
PLR, median (*IQR*)	121.77 (94.84–151.69)	153.56 (106.25–229.68)	<0.001
MLR, median (*IQR*)	0.28 (0.21–0.55)	0.43 (0.28–1.08)	<0.001

### Feature screening

**Figure 1:**
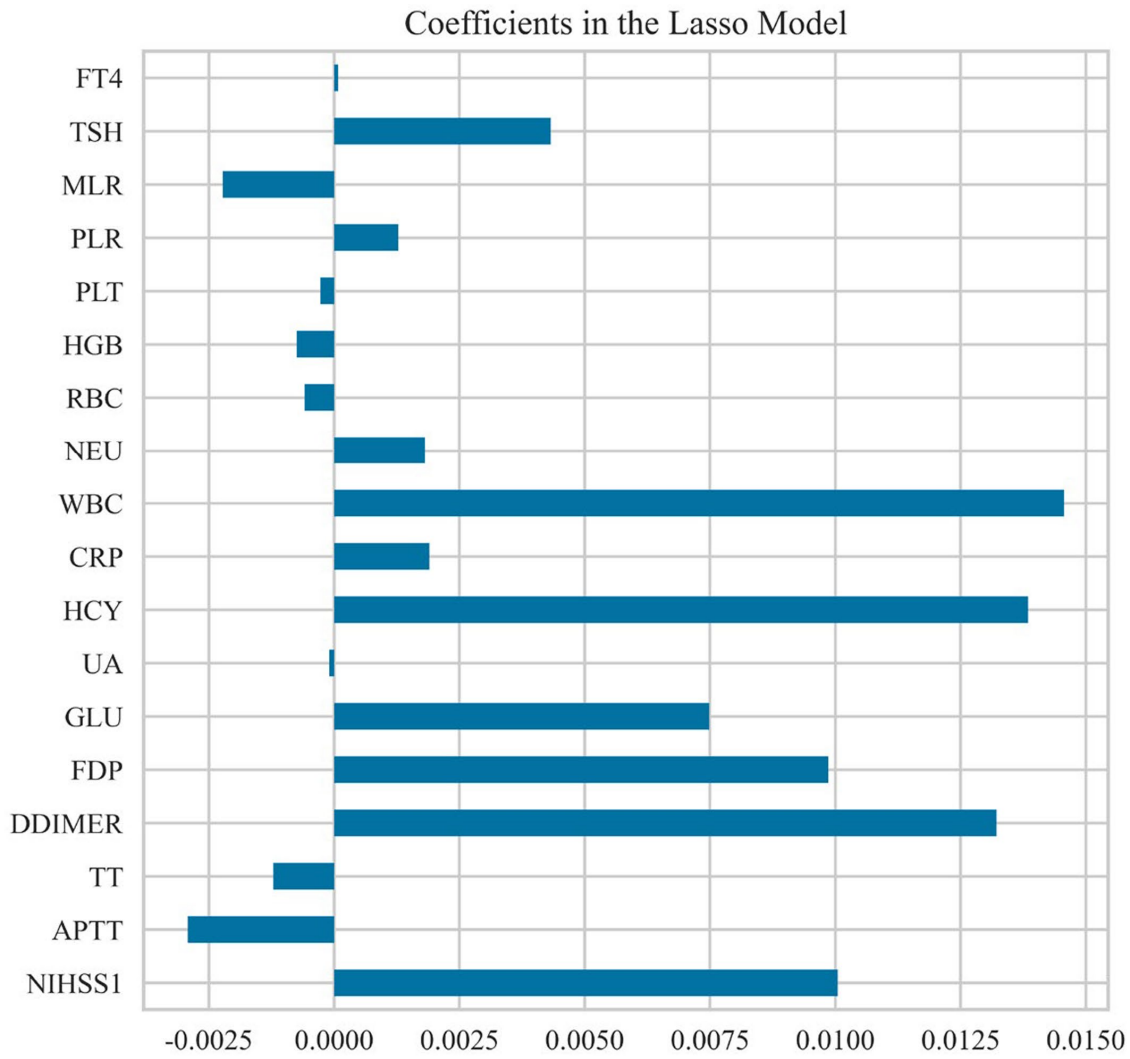
Coefficients in the Lasso model.

In the training dataset, the Lasso CV identified 18 features with non-zero coefficients: NIHSS, APTT, TT, D-Dimer, FDP, GLU, UA, HCY, CRP, WBC, NEU, RBC, HGB, PLT, PLR, MLR, TSH, and FT4, optimizing the regularization parameter *λ* to 0.081. The absolute values of the coefficients were sorted in descending order, revealing WBC, HCY, D-Dimer, NIHSS, FDP, and GLU as the most significant variables. The coefficients of these features are visualized in Figure 1.

### Diagnostic performance of the machine learning models

**Figure 2:**
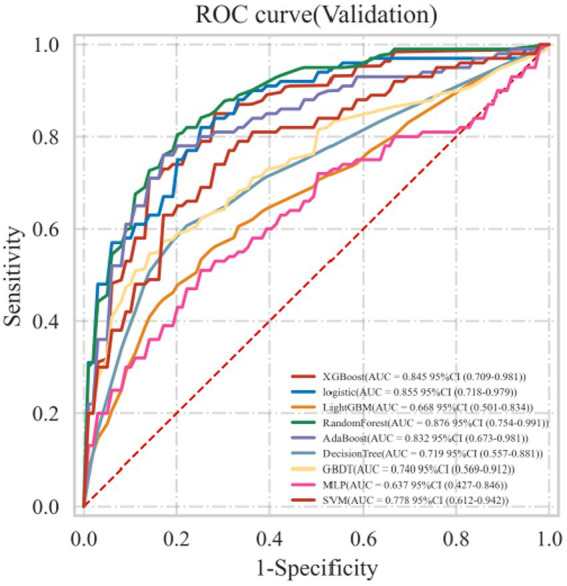
Receiver operating characteristic curve (ROC) for 9 ML models on the validation set.

**Figure 3:**
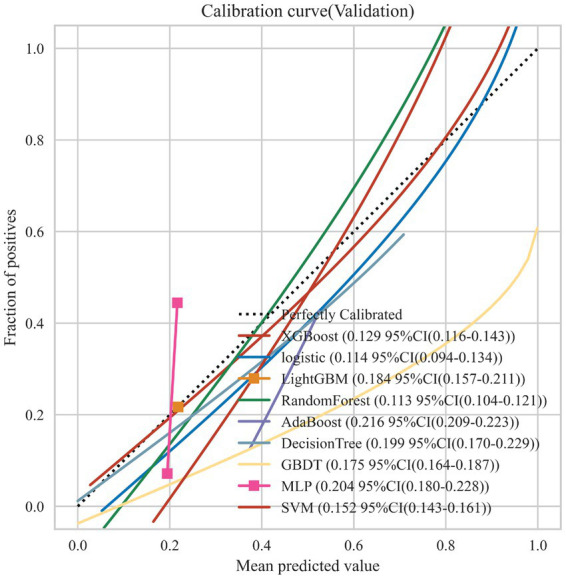
Calibration curves for the 9 ML models on the validation set. The dotted line represents the perfect calibration curve, i.e., the predicted probability matches the true probability perfectly. The numbers in the legend represent the Brier scores of the ML models; the smaller the Brier score, the closer the predicted probability of the ML model is to the true probability.

**Figure 4:**
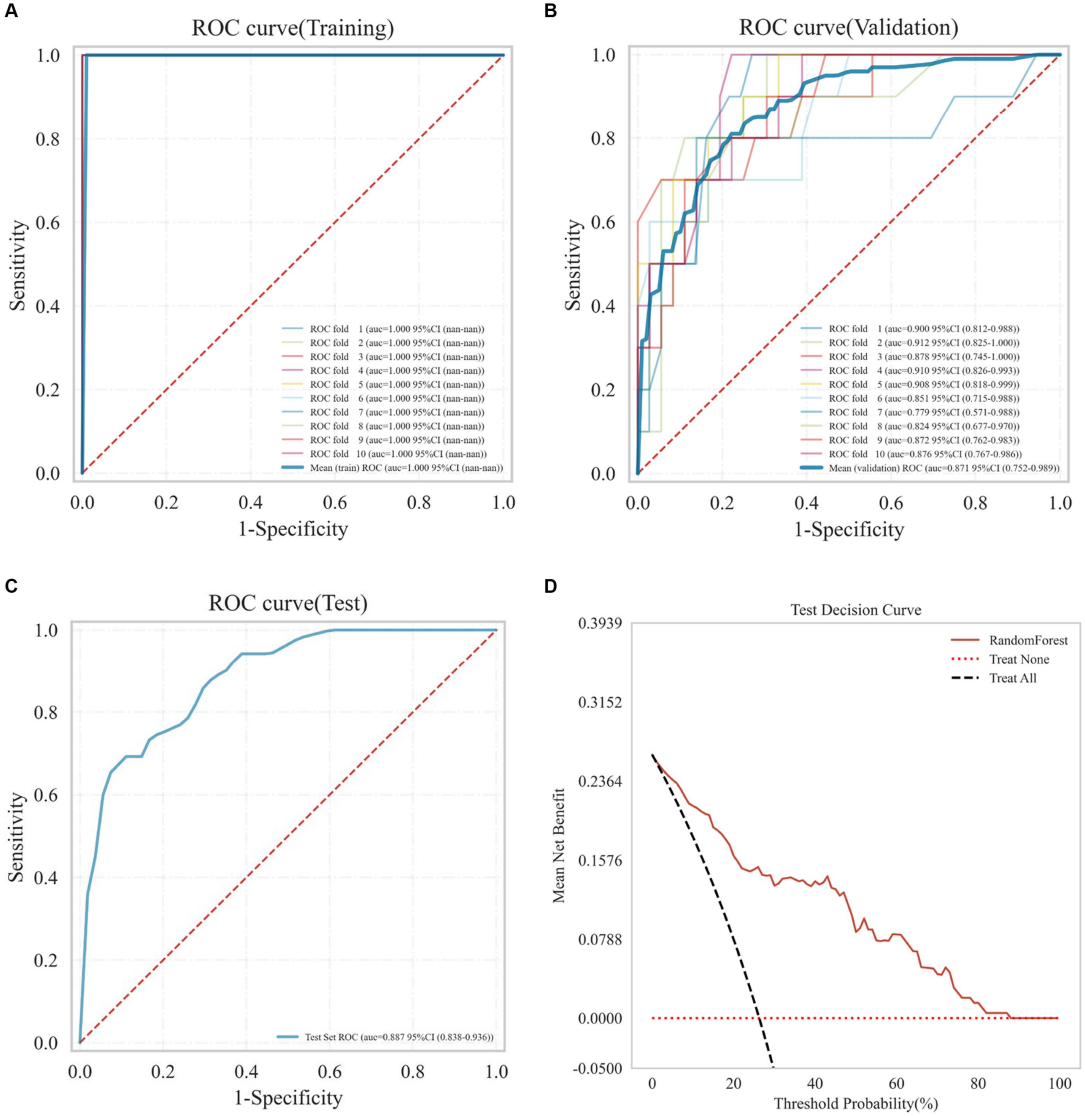
Receiver operating characteristic (ROC) curves of the RF model on the training set **(A)**, validation set **(B)**, test set **(C)**, and the decision curve on the test set **(D)**.

Nine machine learning models were developed using 18 previously identified features, with their performance illustrated in Figure 2, which displays the AUC with 95% Confidence Interval (CI) from 10-fold cross-validation on the validation set. Notably, the RF model demonstrated the highest AUC (0.876 with a 95% CI of 0.754–0.991). Calibration curves (Figure 3) revealed that the RF model exhibited the most accurate alignment between predicted and actual probabilities in assessing the early prognosis of AIS patients, achieving a Brier score of 0.113. This RF model, having shown promising results, underwent further evaluation on a test set, achieving an AUC of 0.852, an accuracy rate of 0.818, a sensitivity of 0.654, a specificity of 0.945, a recall rate of 0.900, and an F1-Score of 0.757. The Receiver Operating Characteristic (ROC) curves for the RF model across training, validation, and test sets are depicted in Figures 4A–C. Additionally, Figure 4D presents the decision curves for the RF model on the test set, highlighting its substantial net clinical benefit across a range of critical risk thresholds. Comprehensive performance metrics of the RF model, including AUC, accuracy, sensitivity, specificity, and recall for training, validation, and test sets, are detailed in Table 2.

**Table 2:** Predictive metrics of RF model on training set, validation set and test set.

Data set	AUC	Accuracy	Sensitivity	Specificity	Recall
Training	1.000	0.997	1.000	1.000	1.000
Validation	0.871	0.833	0.880	0.773	0.867
Test	0.887	0.818	0.654	0.945	0.900

## Discussion

In this study, we developed several machine learning models to predict the early prognosis of AIS patients using general clinical and laboratory data collected within 24 h of admission. Feature selection through the Lasso CV method identified the most critical predictors for early prognosis in AIS patients as WBC, HCY, D-Dimer, baseline NIHSS, FDP, and GLU. Among the nine machine learning models evaluated, the Random Forest model exhibited superior performance in the test set, achieving an AUC of 0.852, an accuracy rate of 0.818, a sensitivity of 0.654, a specificity of 0.945, and a recall rate of 0.900. These findings indicate that RF models utilizing general clinical and laboratory data from the initial 24 h of admission can effectively predict the early prognosis of AIS patients.

ML predictive models leverage algorithms to analyze data and forecast future occurrences or trends. These models have the capability to discern patterns in vast, complex datasets and integrate these patterns in a non-linear and highly interactive fashion (18). The application of machine learning in the medical domain, particularly in identifying risk factors and developing prognostic models for AIS patients, has seen significant growth in recent years, with notable contributions from researchers like Veerbeek et al. (19, 20), Xie et al. (12), Lin et al. (21), Ramos et al. (10), Su et al. (11), and Chen M et al. (22). These studies primarily derive their data from hospital records and publicly accessible databases. For instance, Lin et al. (21) assessed the capabilities of various ML models (SVM, RF, ANN, HANN) in predicting the 90-day mRS outcomes for stroke patients within the Taiwan Stroke Registry. Similarly, Su et al. (11) investigated the predictive performance of 4 ML models (SVM, RF, LGBM, DNN) in forecasting mRS scores and hospitalization outcomes at discharge among 2,780 AIS patients registered in the Chang Gung Healthcare System. Public databases offer vast data volume, broad coverage, and access ease, beneficial for swiftly compiling data required for predictive modeling. Nonetheless, ensuring the quality of data from these sources presents challenges, including risks of inconsistent patient assessment and treatment, incomplete records, and lost follow-up data (22). Predominantly, hospital data-driven studies incorporate imaging, demographic, and clinical information. For instance, Xie et al. (12) utilized imaging data and NIHSS scores to develop prediction models, while Ramos et al. (10) employed imaging and clinical baseline data for AIS prognosis. Common clinical assessments, like routine blood, coagulation, and biochemical tests, serve as conventional, accessible, and cost-effective diagnostics for AIS patients. Yet, the construction of an ML predictive model encompassing demographic, clinical, and 24-h admission blood test data for forecasting 3-month AIS patient outcomes lacks representation in the Chinese population. This study aims to fill this research void.

Previous research primarily focused on the prognostic impact of age (19, 20), atrial fibrillation (4, 23, 24), NIHSS scores at admission (25–27), D-dimer (28, 29), HCY (30–32), and GLU (33–35) on AIS outcomes. In the current study, we employed Lasso CV for feature selection, identifying WBC, HCY, D-dimer, NIHSS, FDP, and GLU as critical variables for developing prognostic models for AIS patients. Notably, WBC and FDP, indicators of the body’s inflammatory response and the fibrinolytic system’s activity, respectively, have been under-explored in AIS prognostication. While WBC has been associated with stroke prognosis and an increased risk of disability at discharge following every 1 × 10^9^/L increment at stroke onset (36), its independent predictive value remains unclear, potentially due to limited sample sizes in previous studies. Similarly, FDP and D-dimer serve as markers for hypercoagulability and hyper-fibrinolysis, pivotal for early diagnosis, monitoring treatment efficacy, and prognosticating thrombogenic conditions. Our findings underscore the importance of including D-dimer and FDP in early prognostic assessments for AIS patients, highlighting their significant role in predicting outcomes.

In this study, we utilized nine machine learning models for analysis, based on selected variables. Of these, the RF model demonstrated superior performance on the test set, achieving an AUC of 0.876 (95% CI, 0.754–0.991). This model provided a significantly better prediction capability. The RF algorithm, known for its robust noise resistance and low susceptibility to overfitting, has found extensive application in healthcare (37). Moreover, several studies employing the RF model for predicting stroke patient outcomes have affirmed its effective predictive power (10, 38, 39).

The current study is constrained by several limitations. Primarily, the modest sample size, despite being derived from two different centers, necessitates a larger dataset for robust validation. Also, this study lacked external validation. In the future, we will conduct a validation study with an external cohort. Moreover, while the study encompasses demographic information, past medical history, blood counts within 24 h of admission, and biochemical assays of AIS patients, it lacks imaging data pertaining to the location and extent of cerebral infarctions. The absence of these imaging details could potentially impact the predictive accuracy of the model. Finally, treatment decision-making is a complex process involving multiple considerations. Our model is just one of many aids whose results should be considered along with clinical judgment and other patient-specific factors, and is intended to provide additional information to support the decision-making process, not to replace professional medical judgment.

## Conclusion

In conclusion, our study illustrates that utilizing demographic data, past medical histories, and 24-h laboratory information upon admission for ML modeling is a feasible approach to predicting the short-term prognosis of patients experiencing their first AIS. Notably, it reveals that the RF model could serve as an effective predictive tool. This finding is crucial for the early identification and prediction of individuals at elevated risk of developing AIS with an unfavorable prognosis. It underscores the importance of prompt and early intervention and treatment to enhance the quality of life for AIS patients.

## Data availability statement

The raw data supporting the conclusions of this article will be made available by the authors, without undue reservation.

## Ethics statement

The studies involving humans were approved by the Medical Ethics Committee of Bengbu Medical University. The studies were conducted in accordance with the local legislation and institutional requirements. The participants provided their written informed consent to participate in this study.

## Author contributions

QH: Writing – original draft, Methodology. G-LS: Data curation, Investigation, Writing – original draft. BS: Formal analysis, Methodology, Writing – review & editing. M-LL: Data curation, Investigation, Writing – review & editing. SZ: Methodology, Writing – original draft. MH: Methodology, Writing – review & editing. F-YH: Writing – original draft.
